# Prevalence of probable sarcopenia in community-dwelling older Swiss people – a cross-sectional study

**DOI:** 10.1186/s12877-020-01718-1

**Published:** 2020-08-26

**Authors:** Julia Wearing, Peter Konings, Rob A. de Bie, Maria Stokes, Eling D. de Bruin

**Affiliations:** 1 Faculty of Health, Medicine and Life Sciences, School for Public Health and Primary Care, Maastricht University, Minderbroedersberg 4-6, 6211 LK Maastricht, The Netherlands; 2 Adullam Stiftung, Mittlere Strasse 15, 4056 Basel, Switzerland; 3 Geriatrische Klinik St. Gallen, Rorschacher Strasse 94, 9000 St. Gallen, Switzerland; 4 School of Health Sciences, University of Southampton, Building 67, Highfield Campus, Southampton, SO17 1BJ UK; 5 Centre for Sport, Exercise and Osteoarthritis Research Versus Arthritis, Nottingham, UK; 6 Department of Health Sciences and Technology, Insitute of Human Movement Sciences and Sport (IBWS) ETH, ETH Zurich, HCP H 25.1, Leopold-Ruzicka-Weg 4, 8093 Zürich, Switzerland; 7 Division of Physiotherapy, Department of Neurobiology, Care Sciences and Society, Karolinska Institutet, Stockholm, Sweden

**Keywords:** Older people, Probable sarcopenia, EWGSOP2, ADL independence

## Abstract

**Background:**

The European Working Group on Sarcopenia in Older People has recently defined new criteria for identifying “(probable) sarcopenia” (EWGSOP2). However, the prevalence of probable sarcopenia, defined by these guidelines, has not been determined extensively, especially in the oldest old. This study aims to determine the prevalence of probable sarcopenia in older, community-living people and its association with strength-related determinants.

**Methods:**

Handgrip strength and reported determinants (age, height, weight, osteoarthritis of hands, medications, fall history, physical activity, activities of daily living (ADL) and global cognitive function) were collected in a cross-sectional study of 219 community-living Swiss people (75 years and over). Probable sarcopenia was estimated based on cut-off values for handgrip strength as recommended by EWGSOP2. Spearman correlations, binary-regression analyses and contingency tables were used to explore relationships between variables.

**Results:**

The prevalence of probable sarcopenia in women (*n* = 137, age 84.1 ± 5.7 years) and men (*n* = 82, age 82.6 ± 5.2 years) was 26.3 and 28.0%, respectively. In women, probable sarcopenia correlated positively with age and falls (r_s_ range 0.332–0.195, *p* < .05), and negatively with weight, cognition, physical activity, using stairs regularly, participating in sports activities and ADL performance (r_s_ range = − 0.141 - -0.409, *p* < .05). The only significant predictor of probable sarcopenia at the multivariate level was ADL performance (Wald(1) = 5.51, *p* = .019). In men, probable sarcopenia was positively correlated with age (r_s_ = 0.33, *p* < .05) and negatively with physical activity, participation in sports and ADL performance (r_s_ range − 0.221 – − 0.353, *p* < .05). ADL performance and age (Wald(1) = 4.46, *p* = .035 and Wald(1) = 6.30, *p* = .012) were the only significant predictors at the multivariate level. Men and women with probable sarcopenia were 2.8 times more likely to be dependent in ADL than those without.

**Conclusion:**

Probable sarcopenia affected one in every four community-living, oldest old people and was independently associated with impaired ADL performance in both sexes. This highlights the importance of detection of handgrip strength in this age group in clinical practice. Although prospective studies are required, independence in ADL might help to protect against probable sarcopenia.

## Background

Sarcopenia is a generalized, progressive muscle disease/disorder [1] that has considerable, negative health-related consequences such as an increased rate of falls and incidence of hospitalization [2]. It is characterized by quantitative and qualitative alterations in muscles that may emerge from middle age onwards [3] and cumulatively occur in people with chronic diseases and an inactive lifestyle [4]. The prevalence of sarcopenia in Europe was between 11 and 20% in healthy men and women aged ≥60 years in 2016 [5, 6] and an accumulated 17% in a recently studied Swiss sample in the Italian speaking part of Switzerland where seven different Sarcopenia definitions were applied [7]. The prevalence significantly increases with age and is expected to rise within the next 30 years, particularly in the highest age groups [5]. Through concomitant diseases, sarcopenia is accompanied by a high personal and social burden when it remains untreated [1]. Sarcopenia can be prevented, delayed and probably counteracted by physical training and nutritional adaptations [4, 8]. However, in clinical practice sarcopenia is not contemplated sufficiently as a determinant of health deterioration [9] notwithstanding the fact that an ICD-10-CM code for sarcopenia was established in 2016. Therefore, sarcopenia can gradually lead to mobility limitations with loss of independence in activities of daily living (ADL) [10] and a decrease of life quality [11].

The European Working Group on Sarcopenia in Older People (EWGSOP) published a consensus paper in 2010 on the definition of sarcopenia in older people based on clinical measures [6]. Pre-sarcopenia was diagnosed by low muscle mass, and sarcopenia was defined by low muscle mass and either low muscle strength (e.g. handgrip strength) or low muscle performance (e.g. low gait speed). Certain cut-off values were set for each assessment. In 2018, the algorithm of clinical measures was updated based on scientific evidence of the previous decade (EWGSOP2) [1] and takes into account the dominant role of muscle strength in age-associated muscle wasting rather than muscle mass. According to the new guidelines, a muscle strength test, such as handgrip strength, is recommended as the initial assessment in detecting sarcopenia, therewith identifying individuals with probable sarcopenia when handgrip strength is < 16 kg for women and < 27 kg for men. Confirmation of the diagnosis sarcopenia is received by an additional detection of low muscle mass or quality. However, low strength on its own is considered enough to initiate interventions [1] since negative consequences of untreated muscle weakness, even without low muscle mass and quality, can be immense for the individual and in terms of health care costs [2]. The evaluation of probable sarcopenia provides important information, relevant for sarcopenia prevention and health promotion.

The handgrip strength test is easily applicable to older adults and provides important information on current and future health status. Low muscle strength increases the probability of mobility limitations [12], impaired performance in daily activities [13–15] and is a marker of frailty [16]. In older people, high grip strength has a protective effect for developing disability [17] while low handgrip strength is a direct, disease-independent predictor of mortality [18, 19]. Therefore, detection of low handgrip strength in older individuals is particularly important to evaluate the status of sarcopenia, which bears relevance for timely initiation of preventive or curative interventions [1]. Since the detection of pre−/probable sarcopenia is now based on muscle strength rather than muscle mass, applying the new guidelines (EWGSOP2) might lead to different population proportions identified with pre-sarcopenia that would qualify for preventive or curative associated care.

To date, information about the prevalence of probable sarcopenia, defined by low muscle strength according to the latest guidelines of the EWGSOP is rare, especially in oldest old men, and not evaluated yet for community living, non-hospitalized women [20–23]. The primary objective of this study was, therefore, to provide data regarding the prevalence of probable sarcopenia in older, community-living women and men in Switzerland. The secondary objective was to evaluate the association between probable sarcopenia and strength-related determinants.

## Methods

### Study design and recruitment

A cross-sectional study, adhering to reporting guidelines [24], was carried out between June 2016 and March 2017 in the Basel and St Gallen urban regions of Switzerland. The aim was to evaluate handgrip strength of community-living Swiss-German older adults. Recruitment was targeted towards people 75 years and over, living independently at their own home. Written information about the study was given to the target population through employees of the study sites (relatives, friends, communities) and organizations for seniors (senior education center and service organization) in the related cities. Interested potential participants were screened for inclusion and exclusion criteria via phone call. Volunteers were included when they had no self-reported upper extremity pain or stiffness on more than 50% of days of the past month, no acute diseases, no injury or surgery of the upper extremity within the past 6 months, were able to understand instructions in German and to sign informed consent. People were excluded when they did not meet the inclusion criteria. Of the potential participants, *n* = 2 had to be excluded due to pain in their hands. The recruitment process was finished when a priori calculated sample size was attained.

### Sample size

Participant numbers (*n* = 219) were estimated a priori based on previously published prevalence data for Swedish older adults [20]. The estimated number would be sufficient to detect a prevalence of 28% at a level of confidence of 0.1 and with 95% precision (d = 0.05).

### Data collection

The assessors were trained and experienced in all testing procedures.

Handgrip strength was evaluated with a calibrated hydraulic hand dynamometer (Jamar®) in accordance with the standardized procedure defined by the American Society of Hand Therapists [25]. In brief, participants were seated with their elbow flexed at 90° and holding the dynamometer in their dominant hand. The handle grip of the dynamometer was standardized to the second smallest grip position. The weight of the dynamometer was counterbalanced by the examiner throughout testing. Based on the cut off value of handgrip strength for probable sarcopenia in women and men, defined by the EWGSOP, the participants were categorized in two groups: category 1 = “probable sarcopenia” (handgrip strength < 16 kg and < 27 respectively) and category 0 = “no probable sarcopenia” (handgrip strength ≥16 kg and ≥ 27 kg).

Factors associated with handgrip strength, as reported in the literature, were also assessed. These were age, height, weight, sedative medication, fall history, osteoarthritis of the hands, global cognitive function, physical activity and basic and instrumental activities of daily living. Data collection is outlined briefly in Table 1, for more detailed explanations we refer to [39].

**Table 1:** Measured variables

Grip strength-related factors	Measure	Rating, categorization
Demographic characteristics [3, 26]	Self-reported age, height and weight	
Sedative medication intake [27]	self-reported	category 0: no sedative medication category 1: ≥ 1 medication
Fall history [28]	self-reported	category 0: no falls category 1: ≥ 1 fall
Osteoarthritis of the hands [29]	self-reported	category 0: yes category 1: no
Global cognitive function [30]	Mini Mental State Exam (MMSE) [31]	points 0–30
Physical activity [26]	Freiburg Questionnaire of Physical Activity [32]	energy expenditure in kcal/week [33, 34]
Basic activities of daily living (ADL) [35]	the Barthel Index [36] Questionnaire as interview	category 0: independent in IADL and ADL highest value in every instrumental and basic activity category 1 (dependent in IADL and/or ADL): 0 points in at least one instrumental activity and less than highest value in at least one basic activity
Instrumental activities of daily living (IADL) [37]	Lawton Scale [38] Questionnaire as interview	

### Statistical analysis

IBM SPSS Statistics, Version 23 was used for statistical analysis. Normal distribution of continuous and categorical variables was tested with the Kolmogorov Smirnov and the Shapiro Wilk test. For further analyses, parametric tests were used for data with a normal distribution and non-parametric tests for non-normal distributed data and categorical variables. Prevalence of probable sarcopenia was estimated by the percentage of individuals with handgrip strength below the cut off value per gender.

Correlations between sarcopenia-categorisations and independent variables were analysed using Spearman’s correlation coefficient and binary-logistic regression. Prevalence of ADL performance in people with probable sarcopenia was evaluated by 2 × 2 contingency tables, calculating the ratio of: number of ADL dependent participants affected by the disease/total cases affected by the disease and number of ADL dependent participants not affected by the disease/total cases not affected by the disease. Pairwise deletion was used in statistical analysis of missing data.

## Results

The prevalence analysis of probable sarcopenia in community-living older people was based on a representative sample of 219 older adults, aged 75 years and over, mean age 83.6 (± 5.6) years (*n* = 137 women, *n* = 82 men) from two urban, German-speaking areas in Switzerland. Mean (SD) handgrip strength in women was 18.4 kg (4.15) and in men 30.9 kg (7.92). The overall percentage of people independent in IADL and ADL was 66%.

### Prevalence of probable sarcopenia

The prevalence of probable sarcopenia, detected by gender-specific handgrip strength cut-off values, was 26.3% in women (*n* = 36) and 28% in men (*n* = 23).

### Associations of probable sarcopenia with factors related to handgrip strength

The variables that were associated with the sarcopenia category (no probable sarcopenia/ probable sarcopenia) varied between sexes. Therefore, further analysis was performed separately for women and men.

#### Correlation analysis

In women, probable sarcopenia correlated positively with age and falls (r_s_ range 0.332–0.195, *p* < .05), and negatively with body weight, cognition, physical activity, using stairs regularly, participating in sports activities and ADL performance (r_s_ range = − 0.141 - -0.409, *p* < .05).

In men, probable sarcopenia was positively correlated with age (r_s_ = 0.33, *p* < .05) and negatively with physical activity, participation in sports and ADL performance (r_s_ range _−_ 0.221 – − 0.353, *p* < .05). ADL performance had the highest correlation of the independent variables in both sexes (Table 2).

**Table 2:** Correlations between sarcopenia category and independent variables in women and men

Independent variables	Women		Men	
	Correlation coefficient	Level of Significance	Correlation coefficient	Level of Significance
Age	r_s_ = 0.332^*^	*p* < .001	r_s_ = 0.33^*^	*p* = .002
Height	r_s_ = − 0.141	*p* = .100	r_s_ = 0.095	*p* = .394
Weight	r_s_ = − 0.176^*^	*p* = .039	r_s_ = − 0.187	*p* = .092
Medication	r_s_ = 0.091	*p* = .291	r_s_ = 0.062	*p* = .578
Falls	r_s_ = 0.195^*^	*p* = .022	r_s_ = 0.136	*p* = .224
Osteoarthritis in hands	r_s_ = − 0.037	*p* = .672	r_s_ = 0.073	*p* = .515
Cognition	r_s_ = − 0.242^*^	*p* = .005	r_s_ = − 0.004	*p* = .975
Physical activity	r_s_ = − 0.323^*^	*p* < .001	r_s_ = − 0.234^*^	*P* = .034
Taking stairs regularly	r_s_ = − 0.224^*^	*p* = .009	r_s_ = 0.001	*p* = .995
Participating in Sport activities	r_s_ = − 0.258^*^	*p* < .001	r_s_ = − 0.221^*^	*p* = .046
ADL performance	r_s_ = − 0.409^*^	*p* < .001	r_s_ = − 0.353^*^	*p* < .001

^*^significant correlation (*p* < .05)

#### Regression analysis

In women, regression analysis showed that the significantly correlating variables age, weight, cognition, physical activity, regularly climbing stairs, participating in sports activities, falls and ADL performance explained whether a participant had probable sarcopenia or not to 32.5% (Nagelkerke’s R^2^ = 0.325, Chi-squared(8) = 33.606, *p* < .000). ADL performance was the only variable significantly impacting on sarcopenia category (Wald (1) = 5.516, *p* = .019) with an Odd’s ratio of 0.263 (Table 3).

**Table 3:** Regression analysis of sarcopenia category by correlated variables in women

Variables	Wald	Degrees of freedom	*p*-value	Exp(B)/ Odd’s ratio	95% Confidence intervall for Exp(B)	
					Lower	Upper
Age	3.211	1	.073	1.093	.992	1.205
Weight	.429	1	.512	.987	.951	1.026
Fall	1.628	1	.202	1.959	.697	5.503
Cognition	.931	1	.335	1.090	.915	1.298
Physical activity	.067	1	.796	1.000	.999	1.001
Participating in Sport activities	.603	1	.438	.592	.158	2.224
Taking stairs regularly	2.208	1	.137	.486	.188	1.259
ADL	5.516	1	.019	.263	.086	.802
Constant	2.725	1	.099	.000		

In men, the significantly correlating variables age, physical activity, participating in sports activities and ADL performance explained the outcome of no probable sarcopenia/ probable sarcopenia to 29.3% (Nagelkerke’s R^2^ = 0.293, Chi-squared(4) = 18.667, *p* = .001. Higher age and lower ADL performance had significant impact on having probable sarcopenia (Wald (1) = 6.298, *p* = .012; Wald (1) = 4.461, *p* = .035), age with an Odd’s ratio of 1.153, ADL performance with an Odd’s ratio of 0.295 (Table 4).

**Table 4:** Regression analysis of sarcopenia category by correlated variables in men

Variables	Wald	Degrees of freedom	*p*-value	Exp(B)/ Odd’s ratio	95% Confidence intervall for Exp(B)	
					Lower	Upper
Age	6.298	1	.012	1.153	1.032	1.289
Physical activity	.432	1	.511	1.000	.999	1.000
Participating in Sport activities	.655	1	.418	.554	.132	2.318
ADL	4.461	1	.035	.295	.095	.916
Constant	5.989	1	.014	.000		

### Prevalence of ADL performance with and without probable sarcopenia

Of the women with probable sarcopenia (*n* = 36), 70% (*n* = 25) were dependent in IADL and/or ADL. In the group of women without probable sarcopenia (*n* = 101) only 25% (*n* = 25) were dependent in IADL and/or ADL. In the 23 men with probable sarcopenia, 57% (*n* = 13) were dependent in IADL and/or ADL, while in the 59 without probable sarcopenia only 20% (*n* = 12) were dependent in IADL and/or ADL. Women and men with probable sarcopenia were 2.8 times more likely to be dependent in ADL than those without (Table 5).

**Table 5:** Prevalence of ADL dependence in people with and without probable sarcopenia

	Women (n)			Men (n)		
	Probable sarcopenia	No probable sarcopenia	Ratio	Probable sarcopenia	No probable sarcopenia	Ratio
IADL and/or ADL dependent (dep)	25	25		13	12	
IADL/ADL independent (indep)	11	76		10	47	
Total	36	101		23	59	
Prevalence (ADL dep/total)	0.694	0.247	2.8	0.565	0.203	2.8

## Discussion

The sample complies with the oldest old population of Switzerland in regard to mean age, gender participation and functional performance in ADL [40]. Handgrip strength of this sample is in accordance with strength values previously evaluated in the German-Swiss population of similar age [41]. Therefore, the study sample can be considered representative of the oldest old population in Switzerland.

This study has applied updated EWGSOP2 guidelines to give insights into probable sarcopenia in community-living, non-hospitalized, oldest old women. Thereby, it provides important health-related data of a group not previously studied. The results demonstrate that a high percentage of community-living older women has such low strength that negative consequences, such as dependence in ADL, are likely to occur when strength decline is not counteracted.

The findings build on existing evidence of probable sarcopenia in oldest old men, in which the prevalence of probable sarcopenia is reportedly 28% [21], to also include new data in oldest old women. In the current study, the prevalence of probable sarcopenia in community-living older people aged 75 years and over was 26.3% in women and 28% in men. These results are in accordance with studies in other countries that assessed people of the same age group [20, 21]. Reiss et al. detected a prevalence of low handgrip strength of 28% in women, and 33% in men in 144 geriatric inpatients (mean age 80.7 years) [21] while Franzon et al. evaluated a 28% prevalence in 287 community-living Swedish men (mean age 86 years) [20]. Two studies of younger populations from Korea and Japan reported lower prevalences of people with low handgrip strength (14.6% in men and women combined [22], 17.2% in men and 10.1% in women [23]). The differences between the previous studies and the present results may be explained by the age differences (mean age 76 years in their studies versus 83 years in the present study) and/or ethnic origin of the study population [22, 23], since handgrip strength declines with age [39] and is reportedly lower in Asian than in European countries [42].

Studies that applied the updated guideline EWGSOP2, as in the present study, but used different measures than handgrip strength for the detection of probable sarcopenia, showed higher prevalences [20, 23]. One study defined male participants (mean age 87 years) as having probable sarcopenia when handgrip strength and/or chair stand test was below cut-off values [20]. This resulted in a prevalence of 73% of which 61% was due to participants who performed poorly in the chair stand test. Another study, applying the same assessment method, also found higher prevalences in comparison to a handgrip strength test alone (23.5% versus 13.7%), even though the participants were younger (mean age 75 years) [23]. Prevalence of probable sarcopenia was shown to be dependent on applied measures of detection in previous literature. However, it is not clear yet which of the strength tests detecting probable sarcopenia is most likely to predict people developing sarcopenia.

However, all studies indicate that handgrip strength below a cut-off value of < 16 kg for women and < 27 kg for men is present in a substantial percentage of the older old population, even in non-institutionalized people. The results have clinical implications since they may increase the awareness of older individuals but also medical doctors and insurance companies about the importance of low muscle strength and the associated high risk of developing sarcopenia. Since probable sarcopenia is a pre-stage of sarcopenia, early detection of low muscle strength is crucial to prevent further decline. These data show that about 27% would benefit from further sarcopenia assessment and specific interventions to avoid the consequences of untreated muscle wasting.

### Associations of probable sarcopenia with factors related to handgrip strength

Factors related to handgrip strength differed between sexes. Low handgrip strength in men was related to higher age, low physical activity and dependence in ADL. In addition, it was also associated with less weight, falls and less cognition in women. ADL performance was independently associated with probable sarcopenia in women and men. Dependence in instrumental and/or basic ADL was 2.8 times more likely in participants with probable sarcopenia than in participants without. Previous cross-sectional studies have shown that handgrip strength, gait speed and chair stand abilities were correlated with restrictions in ADL in older adults [37, 43]. The results of the present study demonstrate the specific risk of ADL dependence, when handgrip strength is below the proposed cut off value by the EWGSOP2.

Low handgrip strength has been shown to be an important indicator of future ADL performance [13–15]. However, it would seem not only important to maintain adequate handgrip strength to ensure independence in daily activity, but also to remain as independent as possible to maintain handgrip strength. The present data may also suggest that independence in ADL execution might help to protect people from probable sarcopenia. A cause-and-effect relationship cannot be drawn by this cross-sectional study but existing prospective studies support this hypothesis, showing that handgrip strength decreases as a consequence of a sedentary lifestyle and a reduced engagement in demanding household activities [17, 44]. Moreover, regular house work and strenuous activities in daily living, such as gardening, have been shown to be protective against dependence in ADL [44, 45]. As performance in ADL as well as muscle strength are modifiable and have the potential to prevent or delay excessive muscle weakness [4, 8], it would be useful to detect decline in muscle strength as soon as possible to ensure timely interventions.

### Limitations

Some limitations of this cross-sectional study should be mentioned. Firstly, the sample included a representative group of 219 community-living people 75 years and over in German-speaking Switzerland. Generalizability of the results to the French-, Italian- and Romansh-speaking parts of Switzerland as well as to other countries is dependent on region-specific variation in handgrip strength. However, given that handgrip strength of adults 75 years and over in German-speaking Switzerland is similar to that of northern European countries, such as Denmark [39], the results of the current study are likely to be representative for older people from other northern and central European countries. Secondly, the subgroup of male participants was relatively small compared to the total population studied but is useful since published data from men > 80 years are rare. Thirdly, determinants of handgrip strength were not exhaustive but limited to key characteristics consistently identified within the literature to explain the variance in handgrip strength. Fourthly, considering that handgrip strength has been shown to be related to body weight [26], categorization into probable sarcopenia-groups based on strength thresholds normalized to body weight might have changed the significance of our findings. A sub-analysis of our results, however, showed that weight did not differ between sarcopenia and ADL subgroups. Hence, normalization of grip strength would not have changed our results. Finally, the rigidity and weight of the Jamar-dynamometer has been suggested to afford difficulties in assessing handgrip strength in very old populations [46]. However, in our study, the weight of the device was supported by the examiner and the handle position was standardized and comfortable for all participants.

## Conclusions and implications

The present study provided new insight into the prevalence of probable sarcopenia detected by low handgrip strength in community-living, non-hospitalized, oldest old women and men, and highlights the importance of detection of probable sarcopenia in clinical practice. With reference to the present data, one in every four community-dwelling people aged 75 years and over in German-speaking Switzerland could benefit from sarcopenia management to prevent negative consequences associated with the condition. Handgrip strength tests should be applied routinely in clinical assessments of older people to detect loss in muscle strength and to initiate timely intervention.

## Data Availability

All data analyzed in this study are included in this published article. The datasets used and/or analysed during the current study are available from the corresponding author on reasonable request.

## Abbreviations

ADL: activities of daily living
dep: dependent
EWGSOP: European Working Group on Sarcopenia in Older People
IADL: instrumental activities of daily living
ind: independent
MMSE: Mini Mental Status Exam
